# Characterisation of the vitreous proteome in proliferative diabetic retinopathy

**DOI:** 10.1186/1477-5956-10-15

**Published:** 2012-03-05

**Authors:** Hao Wang, Le Feng, Jian Wen Hu, Chun Lei Xie, Fang Wang

**Affiliations:** 1 Department of Ophthalmology, Shanghai tenth People's Hospital, Tongji University School of Medicine, 301 Yanchang Road, Shanghai, 200072, China; 2 Tongji University School of Medicine, 1239 Siping Road, Shanghai, 200092, China; 3 Shanghai Applied Protein Technology Limited Company, 500 Caobao Road, Shanghai, 200233, China

**Keywords:** Proliferative diabetic retinopathy, Vitreoretinal diseases, Quantitative proteomics, Marker proteins

## Abstract

**Background:**

Diabetes can lead to serious microvascular complications such as proliferative diabetic retinopathy (PDR), which results in severe vision loss. The diabetes-induced alterations in the vitreous protein composition in diabetic patients with PDR may be responsible for the presence of PDR. The vitreous humour can be utilised in a variety of studies aimed toward the discovery of new targets for the treatment or prevention of PDR and the identification of novel disease mechanisms. The aim of this study was to compare the protein profile of vitreous humour from diabetic patients with PDR with that of vitreous humour from normal human eyes donated for corneal transplant.

**Results:**

Vitreous humour from type 2 diabetic patients with PDR (n = 10) and from normal human eyes donated for corneal transplant (n = 10) were studied. The comparative proteomic analysis was performed using two-dimensional fluorescence difference gel electrophoresis (2-D DIGE). Differentially produced proteins (abundance ratio > 2 or < -2, *p *< 0.01) were identified by matrix-assisted laser desorption ionisation time-of-flight mass spectrometry (MALDI-TOF MS) and MALDI-TOF tandem mass spectrometry. A total of 1242 protein spots were detected on the 2-D master gel of the samples, and 57 spots that exhibited statistically significant variations were successfully identified. The spots corresponded to peptide fragments of 29 proteins, including 8 proteins that increased and 21 proteins that decreased in PDR. Excluding the serum proteins from minor vitreous haemorrhage, 19 proteins were found to be differentially produced in PDR patients compared with normal subjects; 6 of these proteins have never been reported to be differentially expressed in PDR vitreous: N(G),N(G)-dimethylarginine dimethylaminohydrolase 1 (DDAH 1), tubulin alpha-1B chain, gamma-enolase, cytosolic acyl coenzyme A thioester hydrolase, malate dehydrogenase and phosphatidylethanolamine-binding protein 1 (PEBP 1). The differential production of pigment epithelium-derived factor (PEDF) and clusterin was confirmed by Western blot analysis.

**Conclusions:**

These data provide an in-depth analysis of the human vitreous proteome and reveal protein alterations that are possibly involved in the pathogenesis of PDR. Further investigation of these special proteins may provide potential new targets for the treatment or the prevention of PDR.

## Background

Proliferative diabetic retinopathy (PDR), a serious eye complication of diabetes, is characterised by a pathological process that includes capillary occlusion, tissue ischaemia, neovascularisation, increased vascular permeability, and the breakdown of the blood-retinal barrier (BRB) [1]. Subsequently, blindness can result from fibrovascular proliferation, vitreous haemorrhage, tractional retinal detachment, and the development of neovascular glaucoma (NVG) in PDR [2].

Although the management of risk factors, including hyperglycemia, hyperlipidemia, and hypertension, has been shown to ameliorate diabetes-induced vision loss, the exact pathophysiological mechanisms that are involved in this process remain to be elucidated. Whereas the role of high blood glucose has been suggested to be the primary catalyst for the biomolecular and cellular changes seen in the retina, less is known regarding the intraocular biochemical changes associated with the mechanisms that potentially contribute to the pathogenesis of PDR.

Because the vitreous contacts the retina, the physiological and pathological conditions of the retina affect the protein components in the vitreous. The vitreous provides a means of indirectly exploring the events that are taking place in the retina. Previous reports of vitreous proteins in PDR used the conventional enzyme linked immunosorbent assay (ELISA) method [3-5]. In these studies, however, only a certain set of targeted proteins from vitreous samples were identified because the amount of available vitreous was limited, which makes it difficult to evaluate the changes in the vitreous protein profiles and to identify novel marker proteins of PDR pathogenesis. Currently, modern proteomic technologies have the advantage of facilitating the simultaneous analysis and identification of large numbers of proteins. Recent advances in proteome analysis methods have allowed the further exploration and acquisition of vitreous protein profiles [1,6-8]. In the last few years, proteomics has been applied to explore proteins which were differentially expressed in patients with proliferative diabetic retinopathy and in nondiabetic patients [9-14]. Previous studies have identified some proteins that are differentially expressed in the vitreous of PDR patients. Many of the proteins are involved in such processes as angiogenesis, cellular proliferation, acute phase response, vascular permeability changes, and oxygen-induced vessel loss [7,13]. These studies involved the use of vitreous from non-diabetic patients with macular hole (MH) or macular epiretinal membrane (MEM) as control groups. However, these ocular diseases may have affected the vitreous protein profiles. Therefore, in an attempt to obtain "normal control" samples, we used eyes donated for corneal transplant without any known ocular diseases as a control group.

The technique of two-dimensional fluorescence difference gel electrophoresis (2-D DIGE) provides an accurate quantitative comparison of two groups of samples, allowing the identification of proteins whose levels differ significantly between the two conditions [6]. No previous quantitative proteomic comparison of vitreous humour from type 2 diabetic patients with PDR with that from normal human eyes donated for corneal transplant has been reported. We used 2-D DIGE to perform the quantitative proteomic comparison. Matrix-assisted laser desorption ionisation time-of-flight mass spectrometry (MALDI-TOF MS) and MALDI-TOF/TOF MS/MS were used for protein identification; MS/MS techniques provide additional amino acid data for selected peptides and allow for extremely accurate protein identifications [15]. Some of the proteins that were differentially produced in the vitreous from diabetic patients with PDR and from normal human eyes donated for corneal transplant were confirmed by Western blot analysis.

## Results

### 2-D DIGE

**Figure 1:**
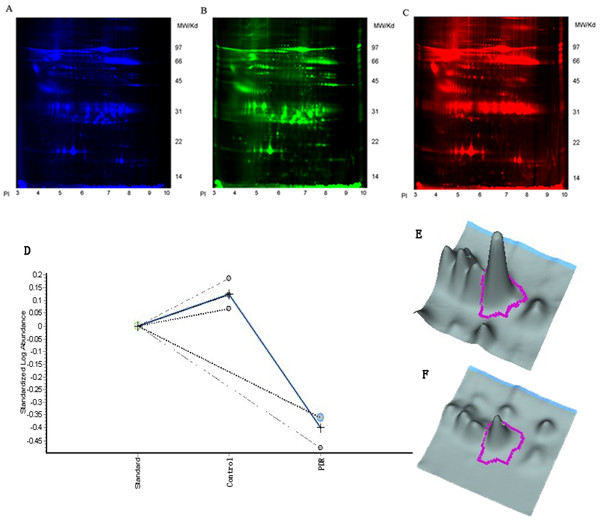
**2-D DIGE images of the master gel (gel 1) and a graph view of spot 599**. (**A**) Cy2 image from the internal standard. (**B**) Cy3 image from the control group. (**C**) Cy5 image from the PDR group. (**D**) The graph view showed the standardised abundance for spot 599 using DeCyder analysis. The blue line links the average abundance values for each group of samples. (**E**) 3D simulation view of DeCyder analysis for spot 599 in the control group. (**F**) 3D simulation view of DeCyder analysis for spot 599 in the PDR group.

The mean protein concentration in the vitreous was significantly higher in patients with PDR than in controls (11.5 vs. 4.32 μg/μl). A representative picture of an overlay of three dye scan images, Cy2, Cy3, and Cy5, is shown in Figure 1. Proteins in the pH range applied in this experiment (pH = 3-10) and in the molecular weight range of approximately 14 to 97 kDa were resolved.

A total of 1242 protein spots were detected in gel 1, 1132 spots were detected in gel 2, and 1028 spots were detected in gel 3. The gel with the greatest number of spots was automatically assigned as the master gel by the DeCyder software. A total of 70 protein spots showed highly significant changes in the expression levels compared with the control group (abundance ratio > 2 or < -2, *p *< 0.01). Each spot of interest was excised from the preparative gel and analysed by MALDI-TOF MS and MS/MS after in-gel tryptic digestion.

### Identified protein lists from MALDI-TOF MS and MS/MS

**Figure 2:**
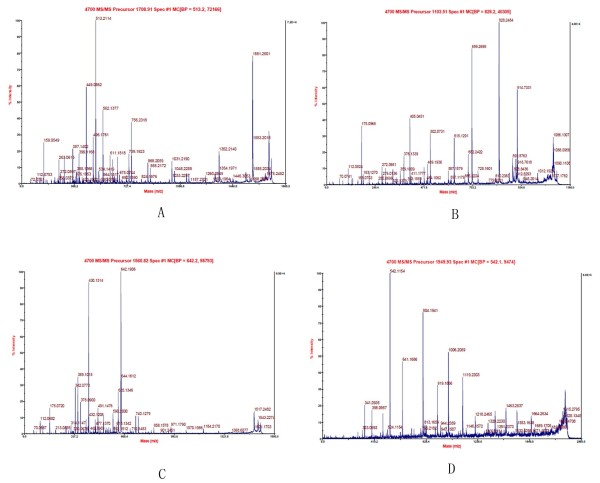
**Identification of proteins by MS and MS/MS**. As an example, the identification of phosphatidylethanolamine-binding protein 1 is represented here. The protein from spot 1010 was excised from the gels and digested with trypsin, and the resulting peptides were analysed using a MALDI-TOF/TOF mass spectrometer. (**A**) The MS spectrum and (**B**) the MS/MS spectrum for the peptide CDEPILSNR. (**C**) The MS/MS spectrum for the peptide LYTLVLTDPDAPSR. (**D**) The MS/MS spectrum for the peptide GNDISSGTVLSDYVGSGPPK. The chart represents m/z (horizontal) versus intensity (vertical). The spectra for the tryptic peptides of PEBP 1 were annotated using the GPS explorer software, v3.6 and the Mascot search engine, v2.1 with the UniprotKB/SwissProt database.

Seventy protein spots were submitted for identification. Among these, 57 spots were matched to the database, and 29 proteins were successfully identified by MALDI-TOF MS and MALDI-TOF/TOF MS/MS (Figure 2). The results of protein identification via MALDI-TOF MS, MS/MS and database research are listed in Table 1. Some of these proteins were identified in several spots.

**Table 1:** Proteins identified from vitreous of PDR

Spot no.(a)	Protein name	Swiss-Prot Access	Biological process	Theoretical		Observed		Mascot Score(b)	Matched peptides	Ratio PDR: C(c)
							
				MW (Da)	PI	MW (kDa)	PI			
437	Ig alpha-1 chain C	P01876	immune response	37630	6.08	63	6.4	281	9	5.45
441	region					63	6.0	366	13	4.29
443						62	6.2	356	11	3.61
467						63	5.8	364	10	3.39
500						70	6.5	143	6	-13.51
486	Tubulin alpha-1B chain	P68363	microtubule-based movement	50119	4.94	67	6.9	100	10	2.36
520	Keratin, type II	P35908	keratinisation	65393	8.07	60	6.6	344	11	2.01
740	cytoskeletal 2					40	6.8	454	13	-7.87
838	epidermal					33	7.6	102	6	3.11
844						32	7.2	72	8	3.14
536	Complement factor I	P05156	immune response	65677	7.72	61	6.7	116	8	2.29
537	Beta-crystallin B1	P53674	visual perception	28006	8.59	62	6.8	101	7	2.89
603						60	5.2	120	7	-2.55
645						45	7.8	127	8	-5.77
573	Hemopexin	P02790	transport	51643	6.55	62	7.1	239	10	3.31
578	Cathepsin D	P07339	proteolysis	44524	6.1	48	5.5	592	19	-3.24
585						49	5.6	361	18	-2.85
580	Pigment	P36955	cell proliferation;	46313	5.97	50	5.7	119	6	-2.92
598	epithelium-derived		negative regulation of angiogenesis;			47	5.8	194	7	-2.96
599	factor (PEDF)		positive regulation of neurogenesis			60	6.2	339	11	-3.36
600	Gamma-enolase	P09104	glycolysis	47239	4.91	60	5.2	625	19	-25.63
639	Zinc-alpha-2-	P25311	fatty acid binding;	33851	6.45	45	5.2	251	12	3.52
654	glycoprotein		ribonuclease activity			43	5.4	150	13	2,99
667						43	5.3	394	17	4.65
689	Cytosolic acyl coenzyme A thioester hydrolase	O00154	hydrolysis	41769	8.85	40	7.6	134	7	-7.16
725	Ig heavy chain V-lll region BRO	P01766	immune response	13218	6.45	42	6.1	101	3	-6.14
726	N(G),N(G)-dimethylarginine dimethylaminohydrolase 1	O94760	arginine catabolic process; signal transduction	31102	5.53	43	5.8	65	5	-6.74
736	Glyceraldehyde-3-phosphate dehydrogenase (GAPDH)	P04406	glycolysis	36030	8.57	37	8.3	308	11	-30.90
755	Clusterin	P10909	apoptosis;	52461	5.89	38	4.8	521	19	-3.43
763			immune response			40	4.5	314	12	-2.56
766						38	5.1	284	15	-2.21
797						34	5.5	114	2	-3.92
800						35	5.9	221	10	-2.24
765	Malate dehydrogenase	P40925	tricarboxylic acid cycle	36403	6.91	38	7.3	199	4	-4.74
794	Keratin, type I	P35527	intermediate filament organisation	62027	5.14	33	5.6	221	16	-2.20
1078	cytoskeletal 9					17	8.5	134	8	-14.34
838	Carbonic	P00918	dehydratase activity;	29228	6.87	32	8.2	223	8	3.11
854	anhydrase 2					32	7.6	136	4	3.54
855			zinc ion binding			33	7.7	390	13	3.13
845	Keratin, type II cytoskeletal 6A	P02538	structural constituent of cytoskeleton	60008	8.09	33	7.4	82	11	3.23
930	Ig lambda-1 chain C regions	P01842	immune response	11230	6.92	30	5.6	95	4	-2.61
942	Prostaglandin-H2	P41222	biosynthesis;	21015	7.66	28	4.9	91	4	-2.53
990	D-isomerase		transport			28	5.1	88	5	-3.32
974	Beta-crystallin S	P22914	structural constituent of eye lens	20993	6.44	28	6.8	147	4	-27.42
978						26	7.5	231	8	-24.32
1019						25	7.2	271	10	-57.43
982	Glutathione peroxidase 3	P22352	hydrogen peroxide catabolic process; oxidation/reduction	25537	8.26	27	5.9	256	6	-2.49
984	Beta-crystallin B2	P43320	visual perception	23365	6.50	27	8.0	113	5	-15.21
1010	Phosphatidylethanolamine-binding protein 1	P30086	protease inhibitor	21044	7.01	28	8.3	437	12	-39.30
1015	Alpha-crystallin B chain	P02511	anti-apoptosis; muscle contraction; intracellular transport; protein folding; protein homooligomerisation; response to heat	20146	6.76	26	7.8	283	9	-26.80
1049	Beta-crystallin A4	P53673	visual perception	22360	5.83	20	6.5	137	5	-5.70
1021	Beta-crystallin A3	P05813	visual perception	25134	5.81	25	6.8	483	12	-109.74
1022						23	7.9	428	11	-149.26
1023						24	7.5	351	12	-233.49
1024	Gamma-crystallin C	P07315	visual perception	20865	6.88	23	7.8	395	12	-147.83

(a) In most cases, multiple spots correspond to the same protein.

(b) Probability-based MOWSE score. Scores higher than 64 indicate the level of statistical significance at *p *< 0.05.

(c) Abundance ratio between the different samples. A ratio > 2 or < -2 indicates statistical significance at *p *< 0.01. The fold-change of downregulation is shown as a negative number, and the fold-change of upregulation is shown as a positive number.

### Validation of selected proteins by Western blot

**Figure 3:**
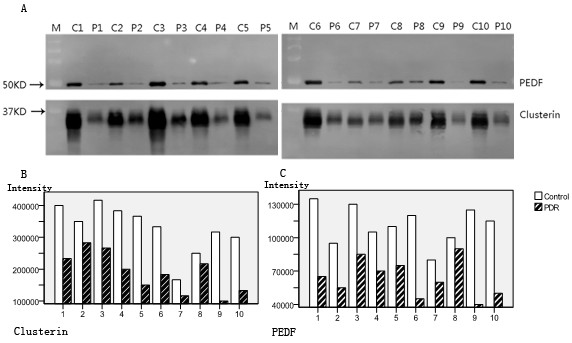
**Validation of the downregulation of clusterin and PEDF in the PDR vitreous by Western blot analysis**. (**A**) Western blot analyses of vitreous samples showing lower levels of PEDF (50 kDa) and clusterin (33 kDa) in the vitreous fluid of ten PDR patients. C (control group), P (PDR group), and M (marker). (**B**) and (**C**) Densitometric analyses of the vitreous PEDF and clusterin levels. Vitreous PEDF and clusterin levels in the PDR group were lower than those of the control group. (*p *< 0.01).

To validate the results obtained in the proteomic study, we selected two candidate proteins involved in angiogenesis and vascular permeability (pigment epithelium-derived factor and clusterin, which were significantly underproduced in the vitreous fluid of PDR patients) to be assessed by Western blot analysis. The band intensity data were generated using the Phoretix 1D. As shown in Figure 3, the PDR groups expressed significantly lower levels of PEDF and clusterin compared with the control when the data were analysed with the Mann-Whitney *U *test (*p *< 0.01).

## Discussion

This is the first report of a comparative proteomic analysis of vitreous humour from type 2 diabetic patients with PDR with that from normal human eyes donated for corneal transplant. From the vitreous samples, we detected more than a thousand spots at a time on a single gel using DIGE; of these spots, 57 showed highly significant changes in the expression level compared with the control group and were successfully analysed as corresponding to peptide fragments of 29 proteins, including 8 proteins that increased and 21 proteins that decreased in PDR. Excluding the serum proteins from minor vitreous haemorrhage, 19 proteins were identified as differentially produced in the vitreous fluid of the PDR patients compared with the vitreous fluid of the normal subjects; 6 of these proteins have never been reported to be differentially expressed in the PDR vitreous: N(G),N(G)-dimethylarginine dimethylaminohydrolase 1 (DDAH 1), tubulin alpha-1B chain, gamma-enolase, cytosolic acyl coenzyme A thioester hydrolase (ACOT1), malate dehydrogenase (MDH) and phosphatidylethanolamine-binding protein 1 (PEBP1). The observed differences in PEDF and clusterin levels by DIGE were further validated by western blot analysis of the samples from each patient, which confirmed the observed differences with good quantitative agreement. The results could not be extrapolated to the other candidates because they have not been verified by Western blot analysis.

Some of the identified proteins appeared at multiple positions on the gels in this and other studies [6,16], which is consistent with the presence of different post-translationally modified forms. Post-translational modifications can change the MW and the pI of proteins, and the various forms of these proteins can migrate to different spots. The quantification of the identified proteins was based on the intensities from multiple spots.

PEDF is produced by the retinal pigment epithelium and serves as a major inhibitor of intraocular angiogenesis. There is growing evidence to suggest that PEDF has a modulatory role in angiogenesis [17]. PEDF alterations in patients with PDR compared with nondiabetic patients are controversial. Some previous studies pointed to reductions in the levels of vitreous PEDF in patients suffering from PDR [6,18-20]. Conversely, elevated levels of PEDF were detected in some studies [13,21]. We have detected reduced levels of PEDF in the vitreous fluid of diabetic patients with PDR by both proteomic analysis and Western blotting. PEDF might be candidate target protein for diabetic retinopathy treatment.

Clusterin is a secreted glycoprotein that has been implicated in a variety of physiological processes, including cell-cell interaction, lipid transport, tissue remodelling, chaperone activity, and apoptosis [22,23]. In recent years, clusterin has been considered a potential diagnostic and prognostic biomarker for several human cancers [24-27]. An and colleagues have demonstrated that clusterin is produced and secreted by retinal pigment epithelial (RPE) cells [28]. Previous studies suggest that during diabetes-induced retinal damage, cytoplasmic clusterin is likely to be associated with protection from cell death, while nuclear clusterin might promote cell death [29]. It is known that clusterin interacts with TGF-β type II receptor (Tβ R II) [30], and TGF-β plays multifunctional roles in regulating the cell cycle, apoptosis, differentiation, and extracellular matrix remodelling [31]. Clusterin is also an important mediator of cell signalling; it can interfere with NF-κB, PI3 kinase, or MAP kinase signalling [32,33], which are associated with cell apoptosis and cell proliferation. In a mouse model of DR, clusterin reduced the leakage from vessels in the diabetic retina, which was accompanied by the restoration of the expression of tight junction proteins [34,35]. These observations suggested that clusterin may play an important role in the prevention of diabetes-induced BRB breakdown. Our data show the downregulation of clusterin in the PDR vitreous, which was confirmed by Western blotting; however, the function of clusterin in the vitreous is not yet clear. PDR is characterised by neovascularisation and enhanced vascular permeability. The proteins involved in the regulation of cell proliferation, apoptosis and BRB breakdown may play important roles in PDR pathogenesis. Therefore, clusterin may contribute to the pathogenesis of PDR, and further studies investigating the precise role of clusterin in diabetic retinopathy are needed.

Carbonic anhydrase (CA) has been identified by proteomic analysis in the vitreous humour from PDR patients; CA production was increased in the vitreous of diabetic retinopathy patients compared with the controls [7,8]. In this study, we have confirmed this finding. The presence of CA was initially thought to be due to retinal haemorrhage and erythrolysis, but subsequent investigation demonstrated that CA is also actively involved in the progression of diabetic retinopathy; CA-1 expression leads to the activation of the contact system, the intrinsic pathway of coagulation, and promotes retinal vessel leakage and intraretinal edema via increased kallikrein activity [8]. The mechanisms involved in the increasing production of CA and the value of CA to potential therapeutic targets require further investigation.

In this study, we identified six proteins that have not previously been reported or described in PDR. It is noteworthy that four of them (DDAH, gamma-enolase, cytosolic acyl coenzyme A thioester hydrolase and malate dehydrogenase) are cellular enzymes, and their levels are all decreased in the vitreous of diabetic patients with PDR, but we do not know whether these changes represent primary causes or consequences of PDR. DDAH is an extremely oxidant-sensitive enzyme [36]. Decreased DDAH expression/activity is evident in disease states associated with endothelial dysfunction and is believed to be the mechanism responsible for the increase in methylarginine levels and the subsequent asymmetric dimethylarginine (ADMA)-mediated endothelial nitric oxide synthase impairment [37,38]. It should be noted that vascular endothelial cells are major targets of hyperglycaemic damage; endothelial dysfunction and reduced levels of endothelial progenitor cells can cause microvascular complications in diabetes mellitus [39,40]. Additionally, oxidative stress appears to play an important role in endothelial dysfunction in diabetes. Therefore, it is thought that the DDAH/ADMA pathway can potentially modulate NO production and endothelial function in PDR. In addition, gamma-enolase has been found in different types of human cancer and is used as a marker for tumoural or cellular damage [41,42]. Gamma-enolase is also used as a marker for neural damage and is a reliable marker for cellular stress during rhegmatogenous retinal detachment (RRD) [43,44]. The changed expression levels of cytosolic acyl coenzyme A thioester hydrolase and malate dehydrogenase in the PDR vitreous could reflect the alterations in glucose and lipid metabolism [45,46]. Moreover, acyl-CoA thioesterases are highly regulated by peroxisome proliferator-activated receptors (PPARs) [47], and PPARγ agonists have shown promise as targets in animal models of proliferative retinopathies [48]. Further studies investigating the role of these enzymes in diabetic retinopathy are needed.

PEBP is a protease inhibitor, and it has been demonstrated to bind to Raf-1 and mitogen-activated protein kinase (MAPK), components of the extracellular signal-regulated protein kinase (ERK) pathway [49]. Aberrant signalling through the ERK pathway could promote cell immortalisation via such mechanisms as telomerase induction, growth factor-independent proliferation, and angiogenesis by the upregulation of proangiogenic factors [50]. Therefore, the lower levels of PEBP detected in PDR patients are perhaps related to neovascularisation and cell cycle progression.

Several types of crystallins, including beta-crystallin S, beta-crystallin B2, alpha-crystallin B chain, beta-crystallin A4, beta-crystallin A3, and gamma-crystallin C, were found in the vitreous humour of both PDR patients and controls. All types of crystallins found in this study were significantly lower in the vitreous humour from PDR patients compared with that from the control subjects, and a previous study has reported that beta-crystallin B2 was identified by MALDI-TOF in normal vitreous [12]. We do not know why the crystallin levels are decreased in the vitreous of diabetic patients with PDR; the biological functions of crystallins are not completely understood. However, it is noteworthy that αA-crystallin and advanced glycation end product (AGE) were highly expressed in human diabetic retinas, and αA-crystallin expression was up-regulated in murine posterior eyecups after recombinant AGE protein was injected into the vitreous of adult murine eyes, αA-crystallin responded to AGE accumulation, which may contribute to the protection of photoreceptors against AGE-related retinal tissue injury [51]. Thus, the mechanisms involved in the intraocular production of crystallins and the role of crystallins in the pathogenesis of PDR require further investigation.

There are two main limitations to this study. First, the gel electrophoresis technique has a number of significant drawbacks. These include its inability to detect low-abundance proteins in the presence of high-abundance proteins or to separate proteins that are too basic, too acidic, too large, or too small [52,53]. Thus, the detection shortcoming may be responsible for our failure to detect vascular endothelial growth factor (VEGF), a key mediator of retinal neovascularisation and vascular permeability in the pathogenesis of diabetic retinopathy [54]. We will apply other proteomic technologies in an effort to pursue this issue. Second, vitreous haemorrhage that occurs in PDR can produce a massive influx of serum proteins. Although we excluded any samples displaying gross vitreous haemorrhage, our results also included some serum proteins resulting from minor vitreal haemorrhage or leakage of serum into the vitreous.

## Conclusions

In this study, we identified 19 proteins in the human vitreous, the expression levels of which were either significantly attenuated or augmented in PDR patients. Clusterin, a potent protective factor in BRB breakdown, was downregulated in the PDR vitreous. Moreover, we identified six proteins, which may be related to endothelial dysfunction, neovascularisation and cell-cycle progression, that have not been previously reported or described in PDR. From the perspective of our study, it is most advantageous and desirable if those differentially expressed proteins also lead toward a more concrete understanding of pathogenesis and therapeutic targets for proliferative diabetic retinopathy.

## Methods

### Study subjects and sample collection

Vitreous fluid was obtained from individuals with proliferative diabetic retinopathy (n = 10) undergoing pars plana vitrectomies at the Shanghai Tenth People's Hospital and the Fudan University Affiliated Eye and ENT Hospital in accordance with the approved Human Discarded Specimen Research Protocols from the institutional review boards. Undiluted vitreous samples (0.5-1.0 ml) were collected at the time of vitrectomy before opening the infusion line. The exclusion criteria were as follows: (1) a history of ocular surgery; (2) gross vitreous haemorrhage or a history of recent vitreous haemorrhage; (3) other ocular diseases such as retinal vein occlusion and age-related macular degeneration; and (4) other systemic diseases aside from diabetes. The control group, consisting of vitreous fluid from normal human eyes without any known ocular diseases (n = 10) that were donated for corneal transplant, were obtained from the Red Cross Eye Bank of Shanghai in China in accordance with the Standardized Rules for the Development and Applications of Organ Transplants. The normal vitreous samples (0.8-1.0 ml) were aspirated with a syringe at the pars plana. The undiluted vitreous samples were collected in tubes and frozen at -80°C until they were required. There was no significant difference in the ages (48.8 ± 11.3 vs. 49.7 ± 8.6; *p *= ns) and the percentage of male subjects (20% vs. 70%, Fischer's exact test, *p *= 0.07) between the diabetic patients and the donors of the control samples.

The research was conducted in accordance with the tenets of the Declaration of Helsinki. The research protocol was approved by the hospital ethics committee, and informed consent was obtained from the patients.

### Vitreous sample preparation

The vitreous samples were lysed at room temperature and subsequently solubilised in lysis buffer (7 M urea, 2 M thiourea, 10 mM DTT, 150 mM Tris, and one complete proteinase inhibitor cocktail tablet per 50 ml lysis buffer) for 30 min. After sonication, the samples were centrifuged at 40000× g for 1 h at 4°C, and the supernatant was subjected to methanol/chloroform precipitation. After the pellets were dried, they were resuspended in lysis buffer. Next, the protein concentrations were determined using BioRad protein assay reagents (BioRad Laboratories, Hercules, CA, USA) and stored at -80°C for subsequent analysis. All chemical reagents were obtained from Sigma Aldrich (St. Louis, MO, USA) unless otherwise noted.

### Two-dimensional difference gel electrophoresis (2-D DIGE)

To reduce the impact of the differences between the individuals, the vitreous samples in the same group were mixed at the same volume before the experiment was performed. The concentration of each sample was adjusted to 5 μg/μl. Equal amounts of each of the samples were pooled to generate the internal standard. The protein samples were minimally labelled with Cy Dye according to the manufacturer's recommended protocols (GE Healthcare). Briefly, the soluble protein samples from the PDR vitreous and control vitreous were labelled with either Cy3 or Cy5 at pH 8-9. Cy2 was used to label the pooled internal standard, which can compensate for gel-to-gel variations. The labelling was performed by adding 400 pmol of the required Cy Dye in 1 μl of anhydrous N,N-dimethylformamide per 50 μg of protein. The labelling reaction was performed on ice in the dark for 30 min, and 1 μl of 10 mM lysine was added for 10 min to terminate the labelling reaction.

Before the 2-D gel electrophoresis was performed, three samples labelled with Cy2, Cy3 and Cy5 were pooled and mixed with rehydration buffer (2 M thiourea, 6 M urea, 4% CHAPS, 20 mM DTT and 0.25% of an appropriate immobilised pH gradient (IPG) buffer). The samples were subsequently applied to IPG strips (13 cm, pH 3-10) for passive rehydration. IEF was performed on an Ettan IPGphor Isoelectric Focusing System(GE Healthcare)at 20°C using the following program: 30 V for 12 h, 500 V for 1 h, 1000 V for 1 h, 8000 V for 8 h, and 500 V for 4 h.

After IEF, the strips were incubated for 15 min at room temperature with equilibration buffer (50 mM Tris, 6 M urea, 30% v/v glycerol, 2% w/v SDS and 2% DTT) followed by a second incubation step in the same buffer solution containing 2.5% iodoacetamide (GE Healthcare) in place of the DTT.

Next, the strips were transferred to the second-dimension 12.5% SDS-PAGE and run on a Hofer SE 600 (GE Healthcare) at 15 mA per gel for 20 min, followed by 30 mA for the remainder of the run until the dye front reached the bottom of the gel. All electrophoresis procedures (labelling, first dimension, and second dimension) were performed in the dark.

After the proteins were separated, all gels were scanned using a UMax Powerlook 2100XL (GE Healthcare). Cy2, Cy3 and Cy5 images were scanned at excitation wavelengths of 488 nm, 532 nm, 633 nm, respectively. We compared the protein abundances between two groups with the ImageQuant and DeCyder v. 5.0 Software (GE Healthcare); three individual 2-D DIGE experiments were performed to obtain consistently detected spots.

### Tryptic in-gel digestion

Protein spots were excised from the preparative gels and destained with 100 mM NH_4_HCO_3 _in 30% ACN. After removing the destaining buffer, the gel pieces were lyophilised and rehydrated in 30 μl of 50 mM NH_4_HCO_3 _containing 50 ng trypsin (sequencing grade; Promega, Madison, WI, USA). After overnight digestion at 37°C, the peptides were extracted three times with 0.1% TFA in 60% ACN. The extracts were pooled and lyophilised. The resulting lyophilised tryptic peptides were stored at -80°C until mass spectrometric analysis. A protein-free gel piece was treated as above and used for a control to identify autoproteolytic products derived from trypsin.

### Protein identification by MALDI-TOF MS and MS/MS

MS and MS/MS spectra were obtained using an Applied Biosystems 4700 Proteomics Analyzer (Foster City, CA, USA). The MALDI-TOF/TOF instrument was operated in a result-dependent acquisition mode. Peptide mass maps were acquired in the positive ion reflector mode (20 kV accelerating voltage) with 1000 laser shots per spectrum. Monoisotopic peak masses were automatically determined within the mass range of 800-4000 Da with a signal-to-noise ratio minimum set to 10 and a local noise window width of m/z 250. The 5 most intense peptides with signal-to-noise ratios exceeding 50 were subjected to MS/MS. The MS and MS/MS spectra were searched against the UniprotKB/SwissProt database (v. 2010.03.02, release number: 15.15/57.15) using the GPS Explorer software, version 3.6 (Applied Biosystems) and the Mascot search engines, version 2.1 (Matrix Science, Boston, MA) with the following parameter settings: one missed tryptic cleavage event allowed, carbamidomethylation set as a fixed modification, oxidation of methionines allowed as a variable modification, peptide mass tolerance set to 100 ppm, fragment tolerance set to ± 0.3 Da, and the minimum ion score confidence interval for MS/MS data set to 95%. Only identified proteins with a protein score confidence index (CI) > 95% were accepted. In the case that shared peptides mapped to more than one protein identifier (ID), the ID with the higher protein score was retained. If multiple IDs had the same protein score, the ID with the larger number of peptides was retained.

### Western blot analysis

To confirm changes in specific proteins, immunoblotting was performed on the 20 vitreous samples used in the DIGE experiments. For normalisation purposes, equal amounts (10 μg) of soluble protein from each vitreous sample were applied to each lane on a 12% acrylamide gel and subsequently electrophoretically transferred to a polyvinylidene fluoride transfer membrane (Hybond-C; Amersham Biosciences Inc., Arlington Heights, IL) at 50 mV for 1.5 h. The membranes were blocked with 5% BSA (w/v) for 1 h at room temperature and incubated overnight at 4°C with primary mouse monoclonal antibodies raised against the full-length recombinant PEDF (50 kDa) and clusterin (33 kDa) of human origin (Santa Cruz Biotechnology, Inc.), diluted 1:3000 and 1:8000, respectively. The blots were washed with TBS-T (0.1% Tween-20 in TBS) three times before incubation with the secondary antibody (horseradish peroxidase-conjugated goat anti-rabbit IgG, 1:5000, Pierce) for 1 h at room temperature. The hybridised membrane was washed in TBS-T buffer and visualised using an ECL Western Blotting Kit (GE Healthcare).

### Statistical analysis

The results obtained for each of the differentially expressed protein spots on the 2-D DIGE gels were analysed statistically using the independent *t *test, and the Western blot intensity data between the groups were compared with the Mann-Whitney *U *test using the SPSS 14.0 statistics package (SPSS, Chicago, IL, USA). A *p *value of less than 0.01 was considered statistically significant.

## Abbreviations

ACOT1: cytosolic acyl coenzyme A thioester hydrolase; BRB: blood-retinal barrier; DDAH1: N(G),N(G)-dimethylarginine dimethylaminohydrolase 1; 2-D DIGE: two-dimensional fluorescence difference gel electrophoresis; MALDI-TOF: matrix-assisted laser desorption ionization time of flight; MDH: malate dehydrogenase; MH: macular hole; PDR: proliferative diabetic retinopathy; PEBP1: phosphatidylethanolamine-binding protein 1; PEDF: pigment epithelium-derived factor; RPE: retinal pigment epithelial; VEGF: vascular endothelial growth factor.

## Competing interests

The authors declare that they have no competing interests.

## Authors' contributions

HW contributed to the conception and design of the experiments, the analysis and interpretation of the data, and the drafting and revising of the manuscript. LF wrote the majority of the manuscript and performed most of the experiments. JWH was responsible for the mass spectrometric analysis and data analysis. CLX participated in sample preparation and performed the statistical analysis. FW is the principal investigator, supervised the project, and provided final additions and edits to this manuscript. All authors read and approved the final manuscript.
